# Early neurological and motor function in infants born moderate to late preterm or small for gestational age at term: a prospective cohort study

**DOI:** 10.1186/s12887-023-04220-w

**Published:** 2023-08-08

**Authors:** Henriette Paulsen, Ulf Wike Ljungblad, Kirsti Riiser, Kari Anne I. Evensen

**Affiliations:** 1 Department of Physiotherapy and Rehabilitation, Vestfold Hospital Trust, Post box 1068, Tønsberg, NO-3103 Norway; 2 Department of Rehabilitation Science and Health Technology, Oslo Metropolitan University, Oslo, Norway; 3 Department of Pediatrics, Vestfold Hospital Trust, Tønsberg, Norway; 4 Department of Clinical and Molecular Medicine, Norwegian University of Science and Technology, Trondheim, Norway; 5 St. Olavs Hospital, Children’s Clinic, Trondheim University Hospital, Trondheim, Norway

**Keywords:** Assessment tools, Low-risk infants, Motor function, Neurological function, Preterm, Small for gestational age

## Abstract

**Background:**

There are inconsistent findings regarding neurological and motor development in infants born moderate to late preterm and infants born small for gestational age at term. The primary aim of this study was to compare neurological and motor function between preterm, term SGA and term AGA infants aged three to seven months corrected age using several common assessment tools. The secondary aim was to investigate their motor function at two years.

**Methods:**

In this prospective cohort study, we included 43 infants born moderate to late preterm with gestational age 32–36 + 6 weeks, 39 infants born small for gestational age (SGA) at term with a birthweight ≤ 10th centile for gestational age, and 170 infants born at term with appropriate weight for gestational age (AGA). Neurological and motor function were assessed once in infancy between three to seven months corrected age by using four standardised assessment tools: Hammersmith Infant Neurological Examination (HINE), Test of Infant Motor Performance, General Movements Assessment and Alberta Infant Motor Scale. The Ages and Stages Questionnaire (ASQ-2) was used at two years.

**Results:**

At three to seven months corrected age, mean age-corrected HINE scores were 61.8 (95% confidence interval (CI): 60.5 to 63.1) in the preterm group compared with 63.3 (95% CI: 62.6 to 63.9) in the term AGA group. Preterm infants had 5.8 (95% CI: 2.4 to 15.4) higher odds for HINE scores < 10th percentile. The other test scores did not differ between the groups. At two years, the preterm group had 17 (95% CI: 1.9 to 160) higher odds for gross motor scores below cut-off on ASQ-2 compared with the term AGA group.

**Conclusions:**

The present study found subtle differences in neurological function between preterm and term AGA infants in infancy. At two years, preterm children had poorer gross motor function. The findings indicate that moderate prematurity in otherwise healthy infants pose a risk for neurological deficits not only during the first year, but also at two years of age when compared with term AGA children.

## Background

It is important to identify neurological deficits and motor impairments, at an early age so that infants requiring individualised follow-up can receive appropriate interventions. Motor impairments in infants at high risk of neurological deficits have been well documented and the highest risks are seen in infants born with low gestational age (GA) in combination with low birth weight [1, 2]. Infants who are born moderate to late preterm (32–36 weeks GA) have received increasing attention, and this group of infants represents 70% of the preterm population [3]. Together with infants born small for GA at term (SGA), they are presumed to be low-risk infants [4], but account for a large percentage of admissions to neonatal units [5].

Low-risk infants are often regarded as equally healthy as term-born appropriate for GA (AGA) infants and there are no recommendations available on the follow-up of low-risk infants in Norway. However, signs of neurological deficits may appear later in childhood, when the more selective movement behaviour enables the child with effective motor function [6, 7]. The literature has yielded inconsistent results regarding motor function in early infancy [8–11]. Studies have reported lower motor performance in infants born SGA [8] and motor delay in moderate to late preterm infants when repeatedly assessed from early infancy to 18 months corrected age [10]. In contrast, a study examining motor function in children with different risk factors from birth, including prematurity and term SGA, found no differences in the beginning of the child’s second year of life compared with term-born AGA children [9]. However, long-term follow-up studies of moderate to late preterm as well as term SGA into later childhood and adolescents have reported an increased risk of motor problems [12, 13].

Valid assessments of early neurological and motor function are crucial for identification of developmental problems. As no single assessment tool considers all of the multiple variables that influence motor development, such as social, environmental and health factors [14], there seems to be a consensus that a combination of assessment tools is better than a single tool when assessing neurological and motor function [1, 14, 15].

The primary aim of this study was to compare neurological and motor function between preterm, term SGA and term AGA infants aged three to seven months corrected age using several, common assessment tools. The secondary aim was to investigate their gross and fine motor function at two years.

## Methods

### Study design

This was a prospective cohort study of infants born moderate to late preterm, SGA at term and AGA at term. The infants were invited together with their mothers as healthy controls, to participate in a study on vitamin B12 status between May 2018 and March 2019 at the Postnatal and Neonatal Unit at Vestfold Hospital Trust, Norway [16]. The infants’ neurological and motor functions were assessed once between the age of three to seven months corrected age using the Hammersmith Infant Neurological Infant Examination (HINE) [17], Prechtl General Movements Assessment (GMA) [18], Test of Infant Motor Performance (TIMP) [19] and Alberta Infant Motor Scale (AIMS) [20]. At two years, the Ages and Stages Questionnaire second edition (ASQ-2) [21] was completed by the parents to assess gross and fine motor function.

### Study population

**Fig. 1:**
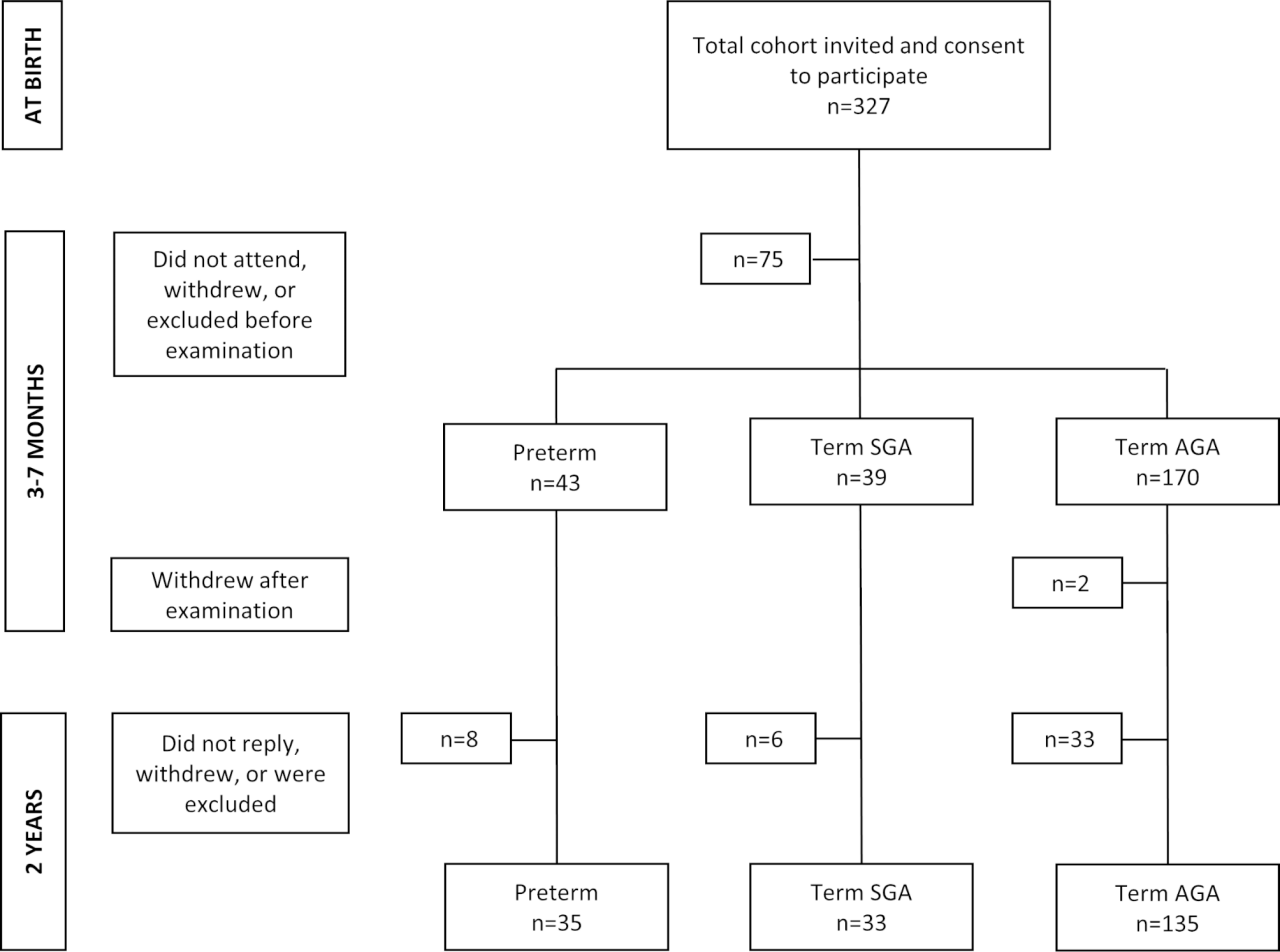
Flowchart of the study population. AGA = appropriate gestational age, Preterm = gestational age 32–36 weeks, SGA = small gestational age

A total of 327 infants were examined for eligibility by a paediatrician at birth (Fig. 1). Inclusion criteria were infants born at GA ≥ 32 weeks, without identified perinatal neurological disease. Forty-nine infants were born at GA 32–36 + 6 weeks (preterm group), 48 were born at GA ≥ 37 weeks and with a birth weight ≤ 10th percentile for GA (term SGA group) and 230 were born at GA ≥ 37 with a birth weight ≥ 10th percentile for GA (term AGA group). Two infants were excluded from the study due to suspected neurological disease, three were excluded due to either cast treatment, infection or maternal illness, and five were excluded due to a vitamin B12 deficiency work-up before assessment. The parents of 65 infants either withdrew or did not attend due to unknown reasons. Thus, 254 infants were examined at 3–7 months. Two term AGA infants were withdrawn by their parents from the study after assessment. At two years of age, parents of eight children in the preterm group, six children in the term SGA group and 33 children in the term AGA group did not reply to the invitation for follow-up assessment or were too old or too young for the ASQ-2 version used.

### Background variables

The following obstetric and perinatal data were retrieved from hospital records: GA, birth weight, length, head circumference, Apgar score at one and five minutes and the mother’s parity. The mother’s country of origin and education were obtained through a questionnaire.

### Procedures

The HINE was carried out by two experienced clinicians: a physiotherapist (HP) and a paediatrician (UWL). The GMA, TIMP and AIMS were carried out by HP, who has experience in the use of TIMP and AIMS, has completed the General Movement Advanced Course and is certified in the GMA. Both examiners were blinded to maternal, obstetric and perinatal characteristics, including GA and birth weight status. The only information known was postmenstrual age. The parents were asked not to inform the examiners of any concerns regarding their infant before the examination was completed and recorded. The examination was performed in a pre-heated room on a mat, or on a wide bench designed for the purpose of examining infants. To obtain a reliable response when being assessed, we strived to have the infant in an active and alert state, as described in the Brazelton Neonatal Behavioral Assessment Scale [22]. The entire examination including all relevant assessments and took approximately 40–50 min to complete. Infants who did not achieve an acceptable state prior to examination were invited to come back for a second consultation, or the examination was declined.

### Outcome measures

All assessment tools used were developed to examine infants at risk of neurological and motor dysfunction [1, 14, 23]. Infants who were too old for the specific assessment tool, were in a non-testable state or had non-completed scoring were excluded from the analysis. We defined neurological and motor function as typical if the score was above recommended cut-offs for each assessment tool.

A standardised infant neurological examination was performed using the HINE [17]. The HINE is divided into three sections: (1) neurological examination, (2) observations of motor milestones and (3) behavioural state. In this study we only used the neurological examination, which gives a numerical score. It consists of 26 items assessing cranial nerve function, posture, movements, tone, and reflexes. The items are scored from zero to three points in 0.5-point steps, with a maximum total score of 78 points. Scores ≤ 10th percentile are regarded as suboptimal. Reference material exists for term infants aged 3–8, 12 and 18 months [24, 25]. The validity of detecting neurological deficits in high-risk [26] and low-risk infants [25] has been proven to be high. The latter study also found excellent inter-rater reliability (ICC 0.953) [25].

We used the GMA for infants up to 20 weeks corrected age to assess neurological function and performed scoring using the ‘Motor Optimality Score for 3- to 5-Month-Old Infants – Revised’ [27]. The GMA is based on a gestalt perception of video-recorded, age-specific normal or abnormal general movements [1, 14, 28]. The infants in the present study were videotaped at the study site for two to five minutes of active wakefulness (not crying and no pacifier), lying in a supine position without any interaction. In healthy infants at three to five months the motor repertoire consists of fidgety movements together with other movements, such as legs lift, foot-to-foot contact, kicking and swiping. Summing the scores of the five subcategories: temporal organisation and quality of fidgety movements, quality of movement patterns, age-adequate movement repertoire, postural patterns and movement character, yields the motor optimality score, ranging from a minimum of five to a maximum of 28 points. A score between 25 and 28 is considered optimal, and scores ≤ 25 indicate suboptimal or reduced motor performance [27]. The GMA has overall demonstrated high validity in detecting severe neurological deficits in high-risk infants [14, 29].

Motor function was assessed using the TIMP for infants up to 17 weeks corrected age. It consists of 42 items, 13 observed and 29 elicited, which include postural change, adaptation to handling, anti-gravity movement, visual reaction, auditory reaction and postural control of the head and body. For the observed items, a score of one is given if present and zero if absent. Each of the elicited items has its own scale varying from one to six points, with a maximal total score of 170 points [19]. According to the normative references, a cut off value − 0.5 standard deviations (SD) below the mean has high sensitivity for detecting developmental problems in high-risk infants [23, 30]. It is considered one of the best tools for discriminating between age-appropriate and delayed motor function in preterm and term-born infants [14, 23].

Motor function was also evaluated using the AIMS, which is an observational scale created to monitor the motor development of children from birth until the acquisition of independent walking [20]. It contains 58 items which assess the control and integrity of antigravity postures organised into four subscales: prone, supine, sitting and standing. The score consists of a dichotomised choice, ‘observed’ (1 point) or ‘not observed’ (0 points). The total score is used to calculate the infant’s age percentile. The AIMS has shown good psychometric properties after the age of four months and high validity for detecting delayed motor function after the age of eight months [14]. A cut-off at the 10th percentile provides the highest validity for identifying delayed motor function in infants aged three to eight months [31].

The ASQ is an age-appropriate developmental screening tool [21]. The second edition (ASQ-2) has been translated into Norwegian [32] and was completed by the parents at two years of age. The questionnaire covers five developmental domains: communication, gross and fine motor function, personal-social functioning, and problem solving. The results of the gross and fine motor domains were used in the present study. The possible score range for each domain is 0–60. According to the manual, we defined motor function as typical if the child scored above the cut-off in the gross and fine motor domains [21]. Cut-off scores are age-dependent: 36.0 and 27.5 points for gross and fine motor at 24 months and 25.0 points at 27 months [33]. The Norwegian version of ASQ-2 has demonstrated satisfactory reliability, but evidence regarding its validity is limited [32, 34, 35]. However, the instrument has the sensitivity to differentiate between preterm and full-term children [34].

### Statistical analysis

Data were registered in EpiData version 4.4 (EpiData Association, Odense, Denmark) and analyses were performed using IBM SPSS version 26 (IBM Corp, New York, USA).

Crude group differences in continuous variables with a normal distribution were analysed with one-way analysis of covariance (ANCOVA) to adjust for age for the HINE, TIMP and AIMS, and with one-way analysis of variance (ANOVA) with post-hoc Tukey for the GMA. The assumption of normal distribution was assessed by visual inspection of QQ-plots of the standardised residuals. Odds ratio (OR) with 95% confidence interval (CI) was calculated as an estimate of the relative risk for a neurological or motor function below cut-off in the preterm and term SGA compared with the term AGA group. A statistical significance level of p < 0.05 was chosen.

We decided a priori to include the infants’ corrected age for term in all regression models at three to seven months, subtracting from the chronological age of examination the number of weeks until the gestational age of 40 weeks. Age correction was not performed at the two-year follow-up according to the ASQ-2 manual [21].

## Results

### Background characteristics

Background characteristics of infants and mothers in the three groups are presented in Table 1.

**Table 1:** Background characteristics of mothers and infants presented as n (%) or median [interquartile range]

		Preterm (n = 43)		Term SGA (n = 39)		Term AGA (n = 170)	
Origin	Norway Europe Non-Europe	31 4 8	(72) (9.3) (19)	28 7 4	(72) (18) (10)	134 22 14	(79) (13) (8.2)
Education	Elementary High school University	0 8 31	(21) (79)	0 18 20	(47) (53)	6 45 118	(3.6) (27) (70)
Parity	1 2 3 or more	14 20 9	(33) (47) (21)	24 14 1	(62) (36) (2.5)	100 50 20	(59) (29) (12)
Sex	female	17	(40)	23	(59)	84	(49)
Apgar scores	5 min	9	[8–10]	9	[9–10]	9	[9–10]
	10 min	10	[9–10]	10	[9–10]	10	[9–10]

AGA = appropriate gestational age, Europe = outside Norway, Preterm = gestational age 32–36 weeks, SGA = small gestational age

### Neurological and motor function in infancy

Mean scores on the neurological and motor assessment tools for the three groups at three to seven months corrected age are shown in Table 2. Preterm infants had lower HINE scores than the term AGA group: mean difference: -1.5 (95% CI: -2.9 to -0.1). None of the infants in any of the groups had absence of fidgety movements on GMA. Mean GMA motor optimality scores in preterm and term SGA infants were below the cut-off for optimal motor performance: 24.5 (95% CI: 23.6 to 25.4) and 24.8 (95% CI: 23.9 to 25.8), respectively. However, they were not statistically significantly different from those of term AGA infants.

**Table 2:** Neurological and motor assessment scores for preterm, term SGA and term AGA infants

Assessment tools	Preterm (n = 43)			Term SGA (n = 39)			Term AGA (n = 170)			
	n	Mean	(95% CI)	n	Mean	(95% CI)	n	Mean	(95% CI)	p
**HINE** Total score^a^	43	61.8	(60.5 to 63.1)^b^	39	62.5	(61.1 to 63.8)	168	63.3	(62.6 to 63.9)	0.099
**GMA** Motor optimality score	31	24.8	(23.9 to 25.8)	28	24.5	(23.6 to 25.4)	86	25.4	(24.9 to 25.8)	0.175
**TIMP** Total score^a^	16	112.4	(107.8 to 117.0)	18	113.8	(109.4 to 118.1)	54	115.2	(112.7 to 117.8)	0.527
**AIMS** Total score^a^	35	17.7	(16.5 to 18.8)	34	18.1	(17.0 to 19.3)	163	17.9	(17.4 to 18.4)	0.862

AGA = appropriate gestational age, AIMS = Alberta Infant Motor Scale, CI = confidence intervals, GMA = Prechtl General Movement Assessment, HINE = Hammersmith Infant Neurological Examination, Preterm = gestational age 32–36 weeks, SGA = small gestational age, TIMP = Test of Infant Motor Performance, ^a^ adjusted for CA at test, ^b^ p = 0.041 vs. Term AGA

Table 3 shows the proportions of infants with scores below cut-off on the different neurological and motor assessment tools. Scores < 10th percentile on HINE were found in 17 (39.5%) of the preterm infants, corresponding to an odds ratio of 5.8 (95% CI: 2.6 to 12.8) compared with the term AGA group. On GMA, 16 (51.6%) of the preterm infants and 14 (50.0%) of the infants born SGA had scores below the cut-off. The odds for scoring below were 0.6 (95% CI: 0.3 to 1.4) and 0.7 (95% CI: 0.3 to 1.5) compared with the term AGA group, respectively, and not significant. There were no differences in proportions scoring below the cut-off for TIMP or AIMS between the groups at three to seven months.

### Motor function at two years

At two years the odds ratio for scoring below the cut-off on ASQ-2 gross motor for preterm children compared to term AGA children was 17 (95% CI: 1.9 to 160) (Table 3).

**Table 3:** Odds for suboptimal scores in preterm and term SGA groups compared with term AGA group

	Suboptimal scores		OR	
	n	(%)	Crude OR	(95%CI)
HINE Total score < 10th percentile Preterm (n = 43) Term SGA (n = 39) Term AGA (n = 168)	17 8 17	(39.5) (20.5) (10.1)	5.8 2.3 1.0	(2.6 to 12.8) (0.9 to 5.8)
**GMA Motor optimality score < 25 points** Preterm (n = 31) Term SGA (n = 28) Term AGA (n = 86)	16 14 34	(51.6) (50.0) (39.5)	0.6 0.7 1.0	(0.3 to 1.4) (0.3 to 1.5)
**TIMP Total score <-0.5 SD** Preterm (n = 16) Term SGA (n = 18) Term AGA (n = 53)	3 3 4	(18.8) (16.7) (7.5)	0.4 0.4 1.0	(0.1 to 1.8) (0.1 to 2.0)
**AIMS Total score < 10th percentile** Preterm (n = 35) Term SGA (n = 34) Term AGA (n = 163)	3 5 18	(8.6) (14.7) (11.0)	0.8 1.4 1.0	(0.2 to 2.7) (0.5 to 4.0)
**ASQ-2 Gross motor score < cut-off at 2y** ^a,b^ Preterm (n = 35) Term SGA (n = 33) Term AGA (n = 135)	4 1 1	(11.4) (3.0) (0.7)	17^e^ 4.2 1.0	(1.9 to 160) (0.3 to 69)
**ASQ-2 Fine motor score < cut-off at 2y** ^c,d^ Preterm (n = 35) Term SGA (n = 33) Term AGA (n = 135)	2 2 1	(5.7) (6.1) (0.7)	8.1 8.6 1.0	(0.7 to 92) (0.7 to 98)

AGA = Appropriate gestational age, AIMS = Alberta Infant Motor Scale, ASQ-2 = Ages and Stages Questionnaires second edition, CI = Confidence interval, GMA = Prechtl General Movement Assessment, HINE = Hammersmith Infant Neurological Examination, OR = Odds ratio, Preterm = gestational age 32–36 weeks, SGA = Small gestational age, TIMP = Test of Infant Motor Performance

^a^cut-off 24 months = 36.0 points, ^b^cut-off 27 months = 25.0 points

^c^cut-off 24 months = 27.5 points, ^d^ cut-off 27 months = 25.0 points

^e^p=0.012

## Discussion

### Main findings

In the present study, we found that infants born moderate to late preterm had poorer neurological function based on the HINE scores compared with term AGA infants. However, motor function assessed with TIMP and AIMS did not differ between the groups. At two years of age, the children born preterm had higher odds for gross motor scores below cut-off on ASQ-2 compared with the term AGA children.

### Methodological considerations

Among the strengths of the present study are the prospective design as well as the use of a broad range of assessment tools to examine neurological and motor function, in a large cohort of healthy, low-risk infants. By recruiting the infants at birth we made certain that the exposure (i.e. risk factors at birth) would be measured before the outcome at three to seven months and at two years of age. However, there is always a possibility that the outcome might be explained by other variables that differ between the groups. A methodological limitation is that the Infant B12 study was not designed to compare the neurological and motor test results of the three groups of infants [16] and was possibly not sufficiently powered to detect differences between the groups. Although preterm and SGA at term infants are representative of low-risk infants, the sample size in each group was small since they were recruited from the general population. This may have limited our power to detect differences [36]. Still, the mean values on the test scores were quite similar between the groups, making type II errors less likely [36], and the differences would probably not be clinically relevant even if they were statistically significant.

The study used well-known and validated assessment tools, and the use of multiple comprehensive assessment tools has proven to increase the possibility of detecting neurological and motor deficits [1, 14]. The HINE was performed independently by the two examiners for 149 infants and demonstrated excellent inter-observer reliability [25]. Utilising several assessment tools in a clinical setting is both time consuming and demanding for the infant and may not be feasible. The GMA, TIMP and AIMS were performed by a paediatric physiotherapist with extensive experience in the use of these assessment tools, and who had knowledge of which items are identical across the tools. This made the assessments more efficient and resulted in less stress for the infants. The HINE, GMA and AIMS have observational sequences that require minimal handling and could thus be performed at the same time. Although the TIMP is the most state-reliable and time-consuming assessment, it consist of items related to the environmental demands placed upon infants during caregiving and could therefore be performed in one sequence [37]. However, the infants who were not in an optimal state at the time of assessment were rescheduled, reducing the chance for biased results [22].

The use of the parent-reported ASQ-2 as a valid measure of gross and fine motor function may be questioned. The ASQ-2 was a priori chosen in the original Infant B12 study in order to assess global development [21]. Compared to validated and structured neurological and motor examination, it has been documented that the ASQ-2 has a limited ability to identify motor difficulties [35].

### Neurological and motor function in low-risk infants

Few studies have investigated presumably healthy infants in studies like the current one. Instead, most studies include infants that are both preterm and SGA, and therefore at an increased risk of impaired neurodevelopment [9, 38]. In the present study, we found that preterm infants had lower total scores on the HINE compared with AGA infants, and they had higher odds of scoring below the 10th percentile. These results are similar to those reported by Chin et al. [39] who found that late preterm infants appeared more immature with discrepancies most apparent in muscle tone and quality of movements on the HINE compared with infants born AGA at term. Likewise, Romeo et al. [40] found lower scores on HINE when comparing preterm infants with different GA at three, six, nine and 12 months corrected age with infants born at term. This could be explained by preterm infants starting extrauterine life with more immature and vulnerable central and sensory-motor systems, which challenge both neurological and motor development [11, 41].

According to our results, both low-risk groups presented with a reduced score on HINE and/or GMA, which are assessments of neurological function [17, 28]. However, all infants presented with normal fidgety movements, which means that they are at very low risk of developing cerebral palsy [28]. Still, a suboptimal score on GMA could indicate subtle neurological impairments in low-risk infants. However, using a 25 points cut-off may be considered too high since a recent study by Kwong et al. [42] found motor optimality score of ≤ 23 predictive for motor and neurosensory impairments in infants born very preterm. Recent studies on the revised GMA motor optimality score have only included infants born very or extreme preterm [42, 43].

In contrast to the HINE and GMA findings, both low-risk groups presented with typical motor function like infants born AGA at term when assessed with TIMP and AIMS. This may be reassuring to both parents and clinicians working with low-risk infants. However, the results at two years showed that the preterm group had higher odds for scoring below cut-off on ASQ-2 gross motor scores compared with the term AGA group. The ASQ-2 results at 2 years of age must be interpreted with caution due to the small number of children scoring below the cut-off. Nevertheless, our results are supported by the findings of Woythaler et al. [44], who assessed a large cohort of late preterm children with the Bayley Scales of Infants Development (Second edition) and found them to have lower scores and increased odds for psychomotor delay at two years compared with term-born children.

The term SGA group presented with typical neurological and motor function in both infancy and at two years. However, neurological and motor function in infants born SGA may not be stable throughout childhood [45].Several studies have reported an increased risk of neurological deficits later in childhood or adolescence [8, 10, 12, 13]. Evensen et al. [12] assessed motor function in SGA adolescents and found that one in six SGA children had motor problems, particularly fine motor problems, at 14 years of age. In line with the present study, term SGA adolescents with motor problems were not identified at the age of one year [46], indicating that longer-term prediction is difficult in these low-risk children.

### Clinical implications

The results of our study should draw attention to the fact that the neurological development of even moderate to late preterm infants may differ from that of infants born AGA at term. The ability to identify infants with typical motor function is important for both health professionals and parents. At two years we did find lower gross motor function in the preterm children when assessed with ASQ-2. However, it would have been interesting to confirm the results in a larger study sample later in childhood and with clinical assessment tools.

The application of assessment tools commonly used in well-baby and follow-up clinics makes this study clinically relevant. The GMA and TIMP are common assessment tools in neonatal units and are used in follow-up clinics along with HINE and AIMS. Ideally, infants with suboptimal neurological and motor function should be identified in early infancy so that follow-up routines can be established accordingly. The HINE was the only assessment tool identifying a difference in neurological function between infants born preterm and AGA at term. Even though a combination of assessment tools has been proven to be more effective in predicting neurological and motor outcomes [1, 14, 15], the use of several assessment tools in a clinical setting is both time consuming and demanding for the infant. Thus, we recommend HINE for the first-line assessment of neurological function in low-risk infants from three months of CA. However, combining HINE with a motor assessment may provide more information about neurological and motor development in infancy.

## Conclusion

The present study found subtle differences in neurological function between preterm and term AGA infants in infancy. At two years, preterm children had poorer gross motor function. The findings indicate that moderate prematurity in otherwise healthy infants pose a risk for neurological deficits not only during the first year, but also motor impairments at two years of age when compared with term AGA children.

## Data Availability

The datasets generated and/or analysed during the current study are not publicly available because permission has not been applied for from neither the participants nor the Ethical Committee but might be available from the corresponding author on reasonable request.

## List of abbreviations

AGA: appropriate for gestational age
AIMS: Alberta Infant Motor Scale
ASQ-2: Ages and Stages Questionnaire second edition
GA: gestational age
GMA: Prechtl General Movement Assessment
HINE: Hammersmith Infant Neurological Examination
SGA: small for gestational age
TIMP: Test of Infant Motor Performance
